# The person-based approach to enhancing the acceptability and feasibility of interventions

**DOI:** 10.1186/s40814-015-0033-z

**Published:** 2015-10-26

**Authors:** Lucy Yardley, Ben Ainsworth, Emily Arden-Close, Ingrid Muller

**Affiliations:** 1 School of Psychology, University of Southampton, Highfield, Southampton, SO17 1BJ UK; 2 Centre for Applications of Health Psychology, School of Psychology, University of Southampton, Southampton, UK; 3 Centre for Behaviour Change, Department of Psychology, Bournemouth University, Bournemouth, UK; 4 Primary Care and Population Sciences, Faculty of Medicine, University of Southampton, Southampton, UK

**Keywords:** Person-based approach, Internet, Qualitative research, Evaluation studies, Feasibility studies, Health promotion, Patient education, Professional education, Behaviour change

## Abstract

**Background:**

This paper provides three illustrations of how the “person-based approach” can be used to assess and enhance the acceptability and feasibility of an intervention during the early stages of development and evaluation. The person-based approach involves using mixed methods research to systematically investigate the beliefs, attitudes, needs and situation of the people who will be using the intervention. The in-depth understanding of users’ perspectives derived from this research then enables intervention developers to design or modify the intervention to make it more relevant, persuasive, accessible and engaging.

**Methods:**

The first illustration describes how relevant beliefs and attitudes of people with asthma were identified from the existing qualitative and quantitative literature and then used to create *guiding principles* to inform the design of a web-based intervention to improve quality of life. The second illustration describes how qualitative “think-aloud” interviews and patient and public involvement (PPI) input are used to improve the acceptability of a booklet for people with asthma. In the third illustration, iterative think-aloud methods are used to create a more accurate and accessible activity planner for people with diabetes.

**Results:**

In the first illustration of the person-based approach, we present the guiding principles we developed to summarise *key design issues/objectives* and *key intervention features* to address them. The second illustration provides evidence from interviews that positive, non-medical messages and images were preferred in booklet materials for people with asthma. The third illustration demonstrates that people with diabetes found it difficult to complete an online activity planner accurately, resulting in incorrect personalised advice being given prior to appropriate modification of the planner.

**Conclusions:**

The person-based approach to intervention development can complement theory- and evidence-based development and participant input into intervention design, offering a systematic process for systematically investigating and incorporating the views of a wide range of users.

**Electronic supplementary material:**

The online version of this article (doi:10.1186/s40814-015-0033-z) contains supplementary material, which is available to authorized users.

## Background

This paper illustrates how the “person-based approach” [1] can be used to assess and enhance the acceptability and feasibility of an intervention during the earliest stages of development and evaluation, in order to maximise the likelihood of a successful outcome when the intervention is subsequently evaluated in a full feasibility study. The aim of the person-based approach is to ground the development of behaviour change interventions in a sensitive awareness of the perspective and lives of the people who will use them, obtained through mixed methods research and particularly iterative qualitative studies. The person-based approach enables the intervention developer to understand how different people in different situations may view and engage with the intervention, which elements may seem particularly relevant and useful to them and which may be rejected—and thus how the intervention can be made more attractive, persuasive and feasible to implement. The first section of this paper briefly introduces the key elements of the person-based approach, and the following sections then illustrate its application in a range of intervention contexts, highlighting methodological issues and demonstrating how crucial insights can be gained from using this approach.

### Core elements of the person-based approach

In-depth qualitative research is a core feature of the person-based approach at the intervention planning and development stage. Theory and evidence from trials of similar interventions can suggest intervention components with the potential to be effective but seldom provide guidance as to which are most important or how best to implement them. Expert and participant involvement can help to provide the patient perspective but is unlikely to be able to represent the views of the entire target user population. Table 1 outlines activities (column 2) suggested as part of the person-based approach to complement activities usually undertaken for intervention planning and development (column 3). At the planning stage, the person-based approach involves carrying out mixed methods research (e.g. interviews, focus groups, observation, questionnaire studies) to systematically investigate the beliefs, attitudes, needs and situation of the people who will be using the intervention in order to identify intervention components that are likely to be necessary, feasible and salient [2, 3]. During development, further mixed methods research (e.g. think-aloud interviews, diary studies, usage analyses) is needed to elicit views of every element of the prototype intervention and allow the researcher to understand the range of ways in which it may be used [4–6]. The intervention is modified on the basis of this research, and then further evaluation is carried out to check whether the changes made have succeeded in making the intervention acceptable, interesting, and easy to use and adhere to.

**Table 1:** Activities relevant to applying the person-based approach at each stage of intervention development and early evaluation

Stage of intervention development and evaluation	Specific person-based approach activities useful at each stage^a^	Other activities relevant to person-based approach normally undertaken as part of intervention development
1. Intervention planning	• Synthesise previous qualitative studies of user experiences of similar interventions • Carry out qualitative research to elicit user views of the planned behaviour changes and intervention (including relevant previous experience, barriers and facilitators)	• Consult experts and stakeholders (e.g. members of user groups, practitioners, purchasers of healthcare services) • Examine relevant theory and evidence from previous trials • Observe real-life context of intended intervention
2. Intervention design	• Use themes arising from the intervention planning stage to identify key issues, needs and challenges the intervention must address • Create guiding principles, comprising: a) Key intervention design objectives b) Key distinctive features of the intervention needed to achieve objectives	• Carry out intervention mapping of behavioural determinants and behaviour change techniques • Create logic model describing hypothesised mechanisms of action of intervention
3. Intervention development and evaluation of acceptability and feasibility	• Elicit and observe user reactions to every intervention element (e.g. using think-aloud techniques), iteratively modifying intervention to optimise acceptability and feasibility • Carry out detailed longitudinal mixed methods case studies of independent intervention usage	• Develop detailed procedures for intervention plus information/advice, manuals, scripts, training, etc. for patients and/or health professionals • Pilot intervention using mixed methods to evaluate acceptability and feasibility

^a^Note that these activities may be carried out iteratively, concurrently or in a different order, not all these activities will be necessary or possible to undertake for every intervention, and this is not intended as an exhaustive list of the types of mixed methods research that could be useful

During the intervention planning phase, we have found it useful to produce *guiding principles* consisting of two elements: *intervention design objectives*, and *key features of the intervention* that can achieve these objectives. The intervention design objectives are based on the key context-specific behavioural needs, issues or challenges that have been identified during the planning stage. The key features of the intervention consist of characteristics of the intervention which should address these objectives. The guiding principles are not exhaustive, and do not replace detailed planning of all the behaviour change elements in an intervention, but can help developers to easily recall and refer to the principal and distinctive features of the intervention which seem particularly important to achieving the intervention objectives.

## Method

In this paper, we provide three brief, previously unpublished, illustrations of how we used the person-based approach for planning and developing interventions for people with asthma and diabetes. These examples were selected to illustrate three different ways in which the person-based approach can contribute to the earliest stages of intervention planning: by providing a process for identifying the key required elements and characteristics of the intervention design, by eliciting crucial early feedback about the acceptability of intervention elements, and by helping to detect and remedy barriers to effective usage of the intervention.

## Results and discussion

### Illustration 1: creating guiding principles to guide intervention development

Creating guiding principles for an intervention is necessarily an iterative process. It is preferable to begin to formulate them at an early stage in order to inform intervention planning—but in the initial stages of intervention planning, it is necessary to base them on incomplete evidence from literature review and primary research. Consequently, the guiding principles must be progressively refined as intervention planning and development proceeds. This case study provides a transparent account of how guiding principles can be formulated from incomplete evidence in the early stages of intervention planning.

There are a number of steps that must first be undertaken to provide the context for developing the guiding principles [1]. The initial stage of planning the development of a digital intervention for the self-management of asthma (My Breathing Matters: “MBM”) was to describe the key objective of the intervention (taken from the proposal): the improvement of patients’ quality of life (QoL) by supporting illness self-management by pharmacological and non-pharmacological means. The next step was to identify the key issues, needs and challenges the intervention must address in order to achieve this objective. For this purpose, we synthesised evidence from both qualitative [Morton K et al: What makes chronic illness self-management interventions acceptable and feasible: a thematic synthesis of users’ experiences, in prep] and quantitative [Mclean G et al: Digital interventions to promote self-management in adults with asthma: systematic review and meta-analysis] systematic reviews of relevant literature, as well as information from mixed methods research undertaken for the development and feasibility testing of relevant previous interventions (*RAISIN*, an online intervention designed to improve medication self-management for patients with asthma [7], and *BREATHE*, a digital video-based intervention designed to help patients with asthma practice breathing retraining [8])*.* Our working summary of evidence used to identify the behavioural needs and issues relevant to developing these particular guiding principles is presented in Additional file 1, which illustrates the range of sources on which the person-based approach can draw in the initial stages—maximising available resources to start to design an effective intervention before later refining acceptability and feasibility using mixed methods research. Qualitative research makes a crucial contribution to this process of identifying user needs and issues relating to the acceptability and feasibility of interventions, as these can generally be elicited best by open-ended, in-depth exploration of the user perspective and the implementation context.

Drawing on these resources, we identified important characteristics of the population (patients with asthma who would be using MBM). With this specific population in mind, we could identify essential relevant issues that the design of the intervention would need to address—for example, particular psychosocial aspects of the population or certain beliefs the users would be likely to hold. For users of MBM, we identified these as the following:

1. Most people with non-optimal asthma control nevertheless do not consider themselves as patients with active asthma [9–11].
2. Therefore, users are not likely to adhere to medication, nor to use an asthma management plan, and may be sceptical of necessity and efficacy of both [9, 12].
3. Other factors contributing to increased symptoms (and reduced QoL) are often not known or acknowledged, particularly (i) anxiety and stress [13] and (ii) lifestyle (e.g. smoking/obesity/avoidance of physical activity [10]).

Having described the intervention objective, the psychosocial characteristics of the target population, and the specific needs, issues and challenges relevant to this population, we were then able to develop the guiding principles themselves (see Table 2). The process of developing guiding principles involves first formulating key intervention design objectives to target each key issue. The next stage is to identify *key intervention features that will address each design objective*. These can include features of the intervention that may have been present in other interventions, as well as features unique to MBM.

**Table 2:** Guiding principles for My Breathing Matters—an intervention to improve functional quality of life of primary care patients with asthma, by helping them to control their condition using pharmacological and non-pharmacological methods

Design objectives that address each key issue	Key intervention features relevant to each design objective
i) To engage people who do not view themselves as having active asthma	• Maintain positive illness context throughout (i.e. promote health rather than manage illness) • Simple, unobtrusive interface to provide optional (and flexible) support only when needed
ii) To persuade and educate users to implement appropriate pharmacological management	• Persuade and educate users regarding the necessity, efficacy and safety of preventative asthma medication • Facilitate easy completion of an action plan with primary care support
iii) To encourage users to employ non-pharmacological methods of improving QoL	• Educate users on benefits and offer psychological methods to improve quality of life (e.g. cognitive behavioural techniques for symptom management) • Provide tailored access and address patient concerns about relevant positive lifestyle changes, such as weight loss if overweight, smoking cessation if current smoker, physical activity if inactive

The key intervention feature*s* are not a comprehensive list of each core ingredient to the intervention; they should be the overarching features around which the bulk of the intervention is developed. The *key features* can prove particularly useful for planning detailed intervention development (i.e. does a particular intervention component have the key elements necessary to achieve a design objective?) as well as for implementing user feedback (i.e. are changes suggested by a user in line with the guiding principles?). Creating the guiding principles required several meetings of the research team, which were valuable at this early stage of planning in terms of allowing team members with different expertise and priorities to highlight aspects of the intervention (and the population that it aims to help) that they considered crucial.

### Illustration 2: using qualitative research to improve the acceptability of an intervention

Qualitative research is invaluable in enabling researchers to improve the acceptability of an intervention. We elicit and observe reactions to every element of the intervention, using think-aloud techniques [14], which enable researchers to observe people using the intervention while saying their thoughts out loud. This has provided valuable insight into people’s views and experiences of an intervention. We then iteratively modify the intervention to improve acceptability.

In this section, we highlight four points (presenting breathing training as something for everyone, the design of the front cover, the importance of continuing with medication and use of personal stories) that arose during the development of the *Breathing Freely* booklet, which was used in BREATHE, a trial of breathing training for asthma [15]. Semi-structured think-aloud interviews were carried out with 29 individuals with asthma to explore their views of breathing training in general and reactions to the booklet (see [8]).

Before the booklet was pilot-tested using think-aloud interviews, we had several meetings with a patient representative. He said he felt that breathing training should be presented as something that is helpful for everyone, rather than as treatment for an illness, as people do not like being labelled. We therefore amended the original booklet to provide the following information on page 5: “Breathing training can be carried out by almost anyone and can help people to feel more comfortable with carrying out their daily activities.” Based on feedback from participants that it would be more helpful to spell out the benefits of breathing training, this was later amended to: “_Q:_ How will doing the breathing retraining benefit me? A: Breathing retraining can be carried out by almost anyone. It is good for everyone, not just for people with asthma, as you learn to breathe more efficiently. Carrying out the breathing retraining may help you to: Feel less wheezy or short of breath, Use your short term ‘reliever’ puffer less often, Do more or walk further before you feel out of breath, Feel more relaxed and Feel more in control of your asthma.”

We changed the front cover image on the booklet several times. Our early images included one of a woman doing yoga, based on feedback from our PPI representative that it would be appropriate to give the booklet a non-medical focus. However, given comments we received from participants that the title did not seem directly related to asthma, we decided to change the picture to something more asthma related. We felt that as we were developing a medical information booklet, a picture of lungs would be appropriate. However, a comment we received from one participant was that: “Yeah I don’t like the picture of the lungs but others might feel differently. But like a friendlier looking booklet, it doesn’t really matter but if you want people to pick it up…” (participant 8). We therefore changed the image to our final image, one of someone blowing a dandelion, which people liked, because they felt it indicated being able to breathe and blow, and therefore demonstrated that breathing training would teach them skills. For example, one participant felt that “…because the picture is really good but I think it gets a good message across that you will have the breath to, you know, to kind of blow on a dandelion…” (participant 16). Similarly, participant 18 said “I think it’s quite good because it indicated being able to breathe and blow and breathing out is quite a lot more difficult for asthmatics than breathing in.”

One other important change we made was to more explicitly highlight the importance of continuing with regular asthma medication. Although our original booklet stated that “… you should continue to take your medications as usual as well as doing the breathing training. The breathing training is not an alternative to your preventer medication”, one participant said that “I think it’s likely that some people, even though it states not to in the booklet, that some people might neglect, if they feel like they’ve increased their breathing capacity, they might neglect to actually use their inhaler … it probably should be stressed a bit more that you really shouldn’t uhh use this as a different option from medication, it’s just an additional thing to help, it’s not a cure or it’s not gonna make it completely better. It’s just gonna relieve some symptoms.” (participant 3). In later versions, we therefore bolded the sentence “You should continue to take your preventer medication as usual”, so participants were in no doubt about this. This was picked up by a later participant who said “You should still take your medication. So it’s not like a kind of alternative to taking medication. You should do it as well.”(participant 6). Qualitative interviews therefore demonstrated the importance of the change we had made.

Another motivational tool included in the booklet was the use of personal stories. We included a story about “Tom”, who practised his breathing exercises while commuting to work on the train, and “Sue”, who used to get out of breath from carrying heavy shopping bags. These stories were well received by the majority of participants, as shown by the following quote: “having the little story in the bubble is nice because, like people can relate to it sort of thing, so they will be like ‘oh yeah I get that’ so it can help me sort of thing, they will think if it can help them it can help me as well.” (participant 4). In line with this quote, participants liked the stories because they could relate to them: “it says Sue’s story about getting out of breath like when shopping in town and carrying heavy bags and like I’ve thought of times when I’ve gone to West Quay shops and have lots of bags and I’m with sort of my housemate or something and she wants to keep going round places and I just get really like: ‘uhh I can’t carry these bags anymore, I can’t walk anymore’, and getting really tired and I sort of…it sort of makes me feel like why can I not keep just walking around but sometimes it just gets umm a little bit stressful … I like her story cause I can kind of understand.” Participants also liked the stories because they showed how much progress the individuals in the stories had made and, by extension, how much it was possible for them to make, as shown here “…Tom’s story, like, shows how much of a benefit [breathing training] is to him. Like going from five seconds to twenty seconds is like a significant amount.” (participant 6).

This example illustrates how in-depth qualitative research provides a unique and crucial opportunity for a diverse sample of users to raise issues that are salient to them and have not been previously considered by researchers. One of our earlier front covers may even have dissuaded participants from using the booklet, and the earlier versions may have inadvertently led participants to stop taking their medication, leading to worsening asthma, but all participants were satisfied with and appropriately motivated by the final version. Qualitative research is thus an essential tool to improve as well as to demonstrate the acceptability of an intervention.

### Illustration 3: using qualitative research to improve the feasibility of an intervention

Qualitative research during intervention development can provide insights beyond assessing the acceptability of an intervention. This section will illustrate how qualitative research methods have been used to improve the feasibility of an intervention.

We developed a web-based intervention (Healthy Living with Diabetes, ISRCTN43587048) to encourage physical activity in people with type 2 diabetes and lower levels of health literacy. A key feature of the intervention was an interactive physical activity planner, designed to help people form concrete and achievable plans for increasing physical activity by building on their current activities. Users were presented with a variety of physical activities and asked on how many days they did each activity in a typical week and for how many minutes each time. The intervention used an algorithm to calculate total minutes of physical activity per week, and users were then presented with tailored feedback informing them whether they need to increase their physical activity levels.

Think-aloud interviews [14] were carried out with a range of people from the target population to assess the acceptability, usability and feasibility of the intervention. Observing participants using the intervention while explaining their thought processes reveals how users enable the researcher to understand and interpret information and instructions. After only a few interviews, it became apparent that users were struggling to complete the physical activity planner accurately; often the context of what they said made it clear that they were misinterpreting instructions or misrepresenting their physical activities. As a result, people with sedentary lifestyles were being presented with intervention pages congratulating them on already being active enough. As we gained insights into how and why people were unable to complete the planner appropriately, we iteratively modified the planner, carrying out further think-aloud interviews repeatedly (with novice users) to check whether the modifications had succeeded in correcting the problems identified.

The original physical activity planner was presented as a single table that required users to enter the number of days and minutes they spent on each activity. Participants were struggling to complete this accurately, and early modifications were made to separate the planner onto two web pages so users could first indicate how many days per week they did certain activities before being asked on a second web page to specify how many minutes they spent on the activity. The original planner also included “climbing stairs” as one of the activities, but early interviews indicated that users were not able to confidently or accurately estimate how much time was spent on this activity. Consequently, the decision was made to remove climbing stairs from the physical activity planner.

**Fig. 1:**
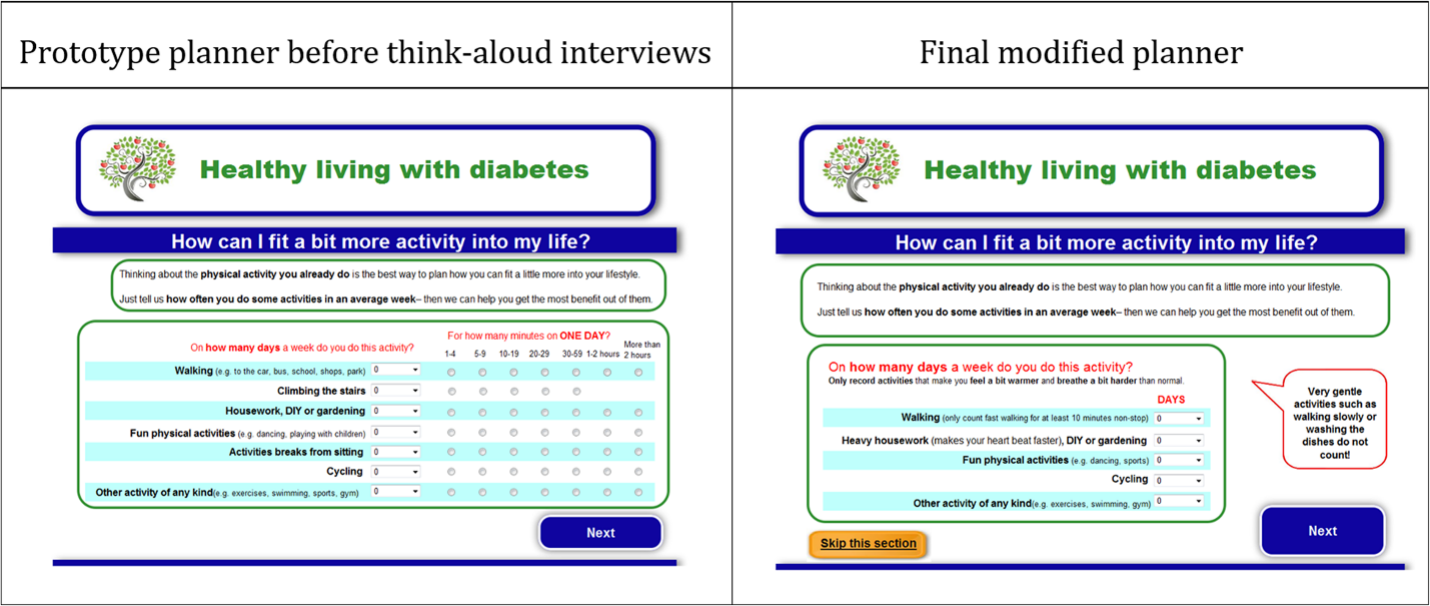
Healthy living with diabetes physical activity planner

Subsequent think-aloud interviews indicated that the usability of the planner had been improved but that users were still vastly overestimating their current levels of physical activity. Further changes were made to the planner, and think-aloud interviews were conducted after each change to assess the impact it was having on people’s ability to complete the planner accurately (see Fig. 1 for an illustration of changes to the planner). One modification was to add general guidance notes, such as “only include activities that make you feel a bit warmer and breathe a bit harder than normal”*.* Another change was to add activity-specific guidance notes, such as “only count fast walking for at least 10 minutes non-stop”. Finally, we added a large, red speech bubble with the text “very gentle activities such as walking slowly or washing the dishes do not count!”*.*

The feasibility of the planner was hugely improved by implementing these changes, and further think-aloud interviews carried out after the final changes had been made indicated that most participants were now able to accurately provide their current physical activity levels. There was still concern that people tended to overestimate their activity levels and hence receive incorrect feedback that they were already sufficiently active. Our final modification was therefore to adapt the algorithm to increase the minimum total amount of physical activity that would lead to this feedback message. Think-aloud interviews were carried out on the final version of the intervention before we were confident that the physical activity planner was feasible and could be implemented. In total, we conducted 35 think-aloud interviews with a range of users from our target population and observed further 4 people completing the physical activity planner.

## Conclusions

The aim of this paper was to illustrate how applying the person-based approach to intervention development can improve the acceptability of interventions and the feasibility of implementing them, from the target users’ perspective. The first two examples highlight the importance of matching the fundamental design of the intervention to the identity and goals of users. Qualitative research and our PPI feedback helped us to understand that most people with asthma like to see themselves as fundamentally healthy, despite intermittent minor symptoms [16], and may therefore be reluctant to engage with an intervention that appears too medical or onerous and hence irrelevant to their situation and needs. Consequently, to increase the acceptability of our asthma interventions we presented them as providing convenient, “light touch” methods to promote and maintain healthy breathing rather than manage long-term illness. Our third illustration demonstrated the necessity of using iterative qualitative research to explore and improve the feasibility of interventions. If we had not done this, then we would not have discovered users’ difficulties reporting their current activity levels correctly, and our intervention would have provided inactive users with tailored advice indicating that they did not need to increase their physical activity levels—the exact opposite of the study aim. These illustrations also highlight the potentially difficult decisions research teams have to make when modifying interventions. Sometimes, decisions can be made on the basis of intervention planning and the guiding principles. Another way we address this is by making iterative changes and assessing the impact of those changes with further qualitative interviews.

This paper provided just three examples of the value of applying the person-based approach, which we have used very extensively [17] and have found essential and effective for ensuring that our interventions are acceptable and feasible. The approach is not intended to replace but to complement and integrate with theory- and evidence-based intervention development—as illustrated by our development of person-based guiding principles for intervention development, which drew on literature review and expert opinion and was carried out alongside a detailed mapping of the complete set of intervention ingredients and associated behaviour change techniques. The second illustration in this paper showed how the person-based approach can also be used to complement participant input into the design of interventions, by specifying a systematic, rigorous process for eliciting and analysing the views of a wide range of users. By using these methods in the earliest stages of intervention development, we are able to ensure that by the time our interventions are evaluated in formal feasibility studies, we have maximised the likelihood that they will be found to be not only acceptable but engaging, feasible to implement and effective.

## Additional file

Additional file 1: **Evidence for key behavioural issues that the intervention is trying to address.** (DOCX 51 kb)
