# Supplementary material for: The person-based approach to enhancing the acceptability and feasibility of interventions
Source: Pilot Feasibility Stud. 2015 Oct 26;1:37. doi: 10.1186/s40814-015-0033-z (PMC5153673; doi:10.1186/s40814-015-0033-z)
Supplement: Additional file 1: — Evidence for key behavioural issues that the intervention is trying to address. (DOCX 51 kb) [file 40814_2015_33_MOESM1_ESM.docx]

**Additional file 1**

**Evidence for key behavioural issues that the intervention is trying to address:**

| **Literature Synthesis: Qualitative Review** | **Literature synthesis: Quantitative Review** | **Existing Research Team Knowledge** | **Evidence used in development of previous interventions (RAISIN)** | **Expert consultation (e.g., HomeBP, specific barriers/facilitators)** |
| --- | --- | --- | --- | --- |

**Paper coding format:**

| **Behavioural Issue** | **Key behaviour** | **Key References** | **Key finding** |
| --- | --- | --- | --- |
| Patients may not have good medication adherence/technique with limited knowledge of necessity/efficacy/safety of medication. | 1. Patients may not have good medication adherence. | **Horne (2006). *Chest*. 130, 1:65S-72S.** [**doi:10.1378/chest.130.1_suppl.65S**](http://journal.publications.chestnet.org/article.aspx?articleid=1210785) | 50% of patients in developed countries do not use medication as recommended – in asthma specifically, 30%-70%  Nonadherence not related to type or severity of disease.  Non-adherence not related to gender/age/SES.  Non-adherence not a trait characteristic. |
|  |  | Sofianou (2013). *J. Gen. Internal Med,* 28, 1: 67:73. [doi: 10.1007/s11606-012-2160-z](http://www.ncbi.nlm.nih.gov/pubmed/22878848) | Illness and treatment beliefs have a strong influence on self-reported medication adherence. |
|  |  | Sumino (2013). *Curr Opin Pulm Med,* 19, 1: 49-53. [doi:10.1097/MCP.0b013e32835b117a](http://www.ncbi.nlm.nih.gov/pubmed/23143198) | Objective measurement of adherence should be implemented where possible. |
|  |  | Lindsay (2013). *Expert Rev Respir Med,* 7, 6: 607-614. [doi:10.1586/17476348.2013.842129](http://www.ncbi.nlm.nih.gov/pubmed/24164107). | Non-adherence is associated with poor baseline asthma control. |
|  |  | Weinstein (2011) *Ann. Allergy, Asthma & Immunology,* 106, 4: 283-291. [doi:10.1016/j.anai.2011.01.016](http://dx.doi.org/10.1016/j.anai.2011.01.016) | Specific adherence strategies (related to cause of non-adherence) required for effective change. |
|  |  | Braido (2015) *Curr Opin Allergy & Clin. Immunol.,*15, 1: 49-55. [doi:10.1097/ACI.0000000000000132](http://ovidsp.tx.ovid.com/sp-3.15.1b/ovidweb.cgi?WebLinkFrameset=1&S=DGDIFPIADMDDKKLFNCKKOGIBLOCAAA00&returnUrl=ovidweb.cgi%3f%26Full%2bText%3dL%257cS.sh.42.43%257c0%257c00130832-201502000-00008%26S%3dDGDIFPIADMDDKKLFNCKKOGIBLOCAAA00&directlink=http%3a%2f%2fgraphics.tx.ovid.com%2fovftpdfs%2fFPDDNCIBOGLFDM00%2ffs046%2fovft%2flive%2fgv025%2f00130832%2f00130832-201502000-00008.pdf&filename=Adherence+to+asthma+treatments%3a++%27we+know%2c+we+intend%2c+we+advocate%27.&pdf_key=FPDDNCIBOGLFDM00&pdf_index=/fs046/ovft/live/gv025/00130832/00130832-201502000-00008) | Physicians believe prescribed dose is taken – causing overprescription, further hindering adherence. |
|  |  | Foster (2014). *J. Allergy & Clin. Imm,* 134, 6: 1260. [doi:10.1016/j.jaci.2014.05.041](http://www.ncbi.nlm.nih.gov/pubmed/25062783) | Inhaler reminders improve adherence in primary care (although may not be reflected in ACQ) |
|  |  | DiMatteo (2012) *Health Psy Rev,* 6, 1: 74-91. [doi:10.1080/17437199.2010.537592](http://www.tandfonline.com/doi/abs/10.1080/17437199.2010.537592) | Cultural norms strongly influence adherence. Information provision is essential but not sufficient to improve adherence. Adherence can be intentional and unintentional. Information should be clear and simple to be understood. |
|  |  | Wilson (2010) *Am Thoracic Coc.* 181, 6: 566-577 doi:[10.1164/rccm.200906-0907OC](http://www.atsjournals.org/doi/abs/10.1164/rccm.200906-0907OC). | Shared decision making improved controller adherence and reduced rescue medication use. |
|  |  | Morrison (in prep) | Self-management DI intervention patients (vs. TAU) improved at managing their own health (PAM), reduced reliever use, improved spirometry and trends to improved AQLQ/ACQ. |
|  |  | Liu (2011) *Eur Resp J.* 37, 2: 310-317. [doi:10.1183/09031936.00000810.](http://www.ncbi.nlm.nih.gov/pubmed/20562122) | Patients in mobile telephone interactive programme increased medication outcome. |
|  |  | Bosley (1995) *Eur Respir J.* 8. 6. 899-904.  [PMID: 7589375](http://www.ncbi.nlm.nih.gov/pubmed/7589375) | Patient self-report and clinican impression of compliance not good predictors of actual compliance.  No difference in anxiety between compliant/non-compliant groups. NC had increased depression. |
|  |  | Rolnick (2011). *Clin Med Res,* 9, 3: 157. | 30% of patients forget to take medication.  16% run out of medication as don’t refll in time.  22% take medication more/less than prescribed in last month.  Barriers cofmmonly noted are an irregular schedule, being too busy.  Facilitators most reported: medications at the same time daily, weekly pill reminder. |
|  | 1. Poor medication technique. | Al-Jahdali (2013). *Allergy, Asthma & Clinl Imm,* 9, 8 : 1-7. [doi:10.1186/1710-1492-9-8](http://www.aacijournal.com/content/9/1/8) | Improper inhaler use is associated with poor asthma control.  Different inhaler types not associated with different technique; device should be selected according to patient preferences. |
|  |  | **Cochrane (2000). *Chest*, 117, 2: 542-550.** [**doi:10.1378/chest.117.2.542**](http://journal.publications.chestnet.org/article.aspx?volume=117&issueno=2&page=542&eaf) | Education programs can improve techniques. Better technique leads to increased lung deposition. |
|  |  | Lenney (2000). *Respir Med,* 94, 5: 496-500. [doi:10.1053/rmed.1999.0767](http://www.sciencedirect.com/science/article/pii/S0954611199907679) | pMDI is most commonly prescribed but not more preferred.  Prescribing patients preferred device improves efficiency but can be cost effective. |
|  |  | Crompton (2006). *Respir Med,* 100, 9: 1479:1494. [doi:10.1016/j.rmed.2006.01.008](http://dx.doi.org/10.1016/j.rmed.2006.01.008) | Prescribing practices vary across Europe.  Medication technique training should be repeated frequently to maintain correct technique. |
|  |  | Yousef (2015). *J Fam Community Med,* 22, 1: 1-7. [doi:10.4103/2230-8229.149567](http://www.ncbi.nlm.nih.gov/pubmed/25657604) | 23% of SA population had good technique knowledge.  Knowledge score significantly better among patients given medication guidelines. |
|  | 1. limited knowledge of medication necessity. | **Federman (2013). *Patient Edu. & Councilling,* 92, 2: 273-278.** [**doi:10.1016/j.pec.2013.02.013.**](http://www.ncbi.nlm.nih.gov/pmc/articles/PMC3720706/) | 36% of patients (older adults from NY) had low health literacy related to asthma.  54% believed they only had asthma when symptoms are present. |
|  |  | Backer (2007). *Allergy & Asthma Proceedings,* 28, 3, 375-381. doi:[10.2500/aap.2007.28.3001](http://dx.doi.org/10.2500/aap.2007.28.3001) | 59% of patients aware that medication dose could be adjusted but only 23% have done so.  All patients wanted more education. |
|  |  | Petrie (2012) *Br. J Health Psychol,*17, 1: 74-84. [doi:10.1111/j.2044-8287.2011.02033.x](http://www.ncbi.nlm.nih.gov/pubmed/22107110) | Intervention group (text-message programme) increased belief in long-term nature of asthma.  Intervention increased perceived necessity of medication and increased perceived control. |
|  | 1. limited knowledge of medication efficacy | George (2014). *J Allergy & Clin. Immunology,* 134, 6: 1252-1259. [doi:10.1016/j.jaci.2014.07.044](http://dx.doi.org/10.1016/j.jaci.2014.07.044) | Complementary/alternative medicine endorsement can lower medication efficacy (and more prevalent in black vs. white adults). CAM/negative inhaler beliefs both related to uncontrolled asthma. |
|  |  | Apter (2003). *J. Allergy & Clin. Immunology,* 111, 6: 1219-1226. [doi:10.1067/mai.2003.1479](http://dx.doi.org/10.1067/mai.2003.1479) | Educational achievement, household income and baseline symptoms associated with adherence.  Favourable attitude to medication associated with greater adherence. |
|  |  | Federman (2013). *Patient Edu. & Councilling,* 92, 2: 273-278. [doi:10.1016/j.pec.2013.02.013.](http://www.ncbi.nlm.nih.gov/pmc/articles/PMC3720706/) | 20% believe their doctor could cure asthma. |
|  | 1. limited knowledge of medication safety | Bender (2005). *Immunol Allergy Clin North Am,* 25, 107-130. [doi:10.1016/j.iac.2004.09.005](http://dx.doi.org/10.1016/j.iac.2004.09.005) | Both patients and physicians report physician is responsible for patient education. 38% of patients report no discussion of medication issues with GPS (need full article). |
|  |  | Horne (1999). *J. Psychosomatic Res,* 47, 6, 555-567. [doi:10.1016/S0022-3999(99)00057-4](http://dx.doi.org/10.1016/S0022-3999(99)00057-4). | Most patients (89%) believed that prescribed medication was necessary for maintain health.  In 17% of patients, safety concern scores exceed necessity scores.  Higher concern scores correlate with lower adherence. |
|  |  | Le (2008). *J. Asthma,* 45:33-37. [doi:10.1080/02770900701815552](http://www.hopkinsmedicine.org/pulmonary/research/adherence_research/document_links/Le_Bilderback_Bender.pdf). | Reduced adherence in ethnic minorities mediated by negative medication beliefs. |
|  |  | Bussey-Smith (2007) *Ann Allergy Asthma & Immunol,* 98, 6 : 07-516. | CAPEPs can improve patient knowledge but effect on objective clinical outcomes ins less consistent |
| They may have limited contact with their primary care team, and may not have a management plan and are less likely to engage in self-monitoring of asthma symptoms (of peak-flow or symptoms). | 1. Limited/poor contact with HCPs | **Cicutto (2014) *J. Continuing Edu. In Health Professions,* 34, 4: 205-214.** [**doi:10.1002/chp.21254**](http://onlinelibrary.wiley.com/doi/10.1002/chp.21254/pdf) | Improvements in patient/HCP encounters lead to significant improvements in asthma control, AAP plan frequency, controller prescription, inhaler technique and appointment arrangement likelihood. |
|  |  | Horne (2007). *BMC Pulm. Med.* 7, 8*.* [doi:10.1186/1471-2466-7-8](http://www.biomedcentral.com/1471-2466/7/8) | Most asthma is managed in primary care.  Asthma control related to patient and HCP behaviours.  GPs need to incorporate assessment of patient perspectives into creation of treatment plan. |
|  |  | Holgate (2006). *BMC Pulm Med,* 6, S2. doi:[10.1186/1471-2466-6-S1-S2](http://dx.doi.org/10.1186%2F1471-2466-6-S1-S2) | Patients have low expectation of receiving appropriate therapy.  Patients have low expectation of having positive encounter with HCP.  HCPs have poor/unstructured communication with patients.  Patients underreport symptoms and severity, leading to misdiagnosis/undertreatment. |
|  |  | **Moffat (2007). *Fam Pract,* 24, 1: 65-70. doi:** [**doi: 10.1093/fampra/cml062**](http://fampra.oxfordjournals.org/content/24/1/65.short) | Patients had positive attitudes towards action plans and guidelines. HCPS reported lacking necessary communication skills, particularly in non-medical areas. |
|  |  | Backer (2012). *Respir. Med,* 106, 5: 635-641. [doi:10.1016/j.rmed.2012.01.005](http://dx.doi.org/10.1016/j.rmed.2012.01.005) | When systematic approach to HCP/asthma management appointment was introduced (used GINA guidelines) asthma control improved significantly. |
|  |  | Moffat (2006). *Prim Care Respir J,* 15, 3: 159-165. [doi:10.1016/j.pcrj.2006.02.006](http://www.ncbi.nlm.nih.gov/pubmed/16757396) | P articipants with severe and/or difficult asthma normalised control issues, were reluctant to discuss non-medical factors with healthcare professionals (HCPs), reported poorer communication with HCPs, and were reluctant to raise relevant but non-medical factors in the consultation |
|  |  | Tan (2009). *Singapore Med J.* 50, 2: 160-164.  <http://europepmc.org/abstract/med/19352577> | HCPs prescribing behaviour varies according to medical training and drug costs in context of local healthcare system. |
|  |  | Foster (2005) *Prim Care Resp J,* 14, 3: 154-60. [PMID: 16701715](http://www.ncbi.nlm.nih.gov/pubmed/16701715) | Patients found nurse specialists approachable and informative but found information from multiple clinicians confusing. |
|  |  | Pinnock (2005) *Prim Care Resp J.* 14: 42-46  [doi:10.1016/j.pcrj.2004.10.002](http://www.nature.com/articles/pcrj2004102) | Telephone consultations convenient for well-controlled asthma; in depth reviews if more severe symptoms. Mobile consultations viewed as lacking a human edge. |
|  |  | Young (2012) *Telemed J E Health*, 2, 3: 49.  doi:[10.1089/tmj.2011.0194](http://dx.doi.org/10.1089%2Ftmj.2011.0194) | 11 of 15 ppnts described telephone interaction with pharmacist as helpful.  Time was considered a barrier although didn’t actually provide any obstacle.  Participants positively viewed opportunities for immediate feedback. |
|  |  | Kerr (2010) *J Med Internet Res,* 12, 4: e56. | Self-management intervention. Range in information amount that patients wanted; related to disease symptom characteristics (recent hospitalization = more awareness).  internet access/familiarity was a barrier to use.  Participant perception of information reliability depended on need and confidence in computers. |
|  |  | Hartmann (2007) *J Med Internet Res.* 9, 1: e3.  DOI: [10.2196/jmir.9.1.e3](http://doi.org/10.2196/jmir.9.1.e3) | Interactive website gave tailored feedback about condition and suggested questions for user to ask physician.  Patients who used website had positive shift in attitude to physicians.  Use of website prompted patients to become more involved in asthma care. |
|  |  | De Jong (2014) *J Med Internet Res.* 16, 1: e19 DOI: [10.2196/jmir.3000](http://doi.org/10.2196/jmir.3000) | Patients are willing to participate and to take initiative to discuss health issues with providers using asynchronous communication. |
|  | 1. Lack of action plan. | Rank (2008). *Mayo Clin Proc,* 83, 11: 1263-1270. [doi:10.4065/83.11.1263](http://dx.doi.org/10.4065/83.11.1263). | Action plans vary widely in readability and usability.  Action plans should clearly define decision points, expected response and expected time of response.  Action plans should include self-monitoring and treatment. |
|  |  | Sarver (2009). *J Am Acad Nurse Pract,* 21, 1: 54-65. [doi:10.1111/j.1745-7599.2008.00375.x](http://onlinelibrary.wiley.com/doi/10.1111/j.1745-7599.2008.00375.x/full). | Action plans should incorporate combination of pharmacologic and non-pharmacologic treatment modalities. |
|  |  | **Ring (2011). *Patient Edu. & Counselling,* 85, 2, 131-143.** [**doi:10.1016/j.pec.2011.01.025**](http://dx.doi.org/10.1016/j.pec.2011.01.025)**.** | Patient illness beliefs must fit within action plan in order for maximum use.  Action plans must be developed in conjunction with HCP.  Patient/carers perceive themselves as capable of disease management, independent of HCP views. |
|  |  | Roy (2011). *Cochrane Coll.* [doi:10.1002/14651858.CD009479](http://onlinelibrary.wiley.com/doi/10.1002/14651858.CD009479/full). | Need for review of asthma self-management schemes and effect on asthma outcomes.  Need for determining of specific characteristics. |
|  |  | Cross (2014). *Cana Resp. J.* 21, 6, 351-356. doi: [PMCID:PMC4266154](http://europepmc.org/articles/PMC4266154) | Only 1 in 10 AAPs written in ED admissions. Increased frequency of AAP related to improved asthma outcomes. |
|  |  | Baptist (2013) *J Am Geriatr Soc,* 61, 5: 747-73.  [doi: 10.1111/jgs.12218](http://www.ncbi.nlm.nih.gov/pubmed/23617712) | mAQLQ significantly higher in intervention group after 1,3,12 months after 6 session program.  ACQ better in intervention program group after 12 months. |
|  |  | Gupta (2012) *Ann Allergy Asthma Immunol,* 108, 4: 260-265. [doi: 10.1016/j.anai.2012.01.018](http://www.ncbi.nlm.nih.gov/pubmed/22469446) | Studies evaluating effectiveness of action plans may not be directly comparable.  Visual design may affect usability, uptake and effectiveness. |
|  |  | Licskai (2013), *Can Respir J,* 20, 4: 301-306. [doi: 10.1016/j.anai.2012.01.018](http://www.ncbi.nlm.nih.gov/pubmed/22469446) | Large majority confirmed ease of use, clarity and timeliness as key factors. |
|  | 1. No self-monitoring of peak flow. | Self (2014). *J Asthma,* 51, 6: 566-572. [doi:10.3109/02770903.2014.914218](http://www.ncbi.nlm.nih.gov/pubmed/24720711). | Monitoring PEF is part of national guidelines for asthma management.  Many patients do not use PFMs properly.  Clinicians should regularly observe PFM use to correct use. |
|  |  | McGrath (2001). *J Clin Pharm Ther*, 26, 4, 311-317. [doi:10.1046/j.1365-2710.2001.00374.x](http://onlinelibrary.wiley.com/doi/10.1046/j.1365-2710.2001.00374.x/full) | No obvious advantage of PFM over symptom monitoring – improvements in both groups.  Patients can underestimate degree of airflow obstruction.  PFM may be appropriate for patients who have difficulty identifying symptom worsening. |
|  | 1. No/poor self-monitoring of subjective symptoms. | Bheekie (2001). *J Clin Pharm & Therapeutics,* 26, 4, 287-296. [doi:10.1046/j.1365-2710.2001.00361.x](http://onlinelibrary.wiley.com/doi/10.1046/j.1365-2710.2001.00361.x/full). | Patients applying symptom monitoring tend to underestimate severity of condition and use medication appropriately.  PEFR self-monitoring proved to be more useful than symptom self-monitoring. |
|  |  | Pinnock (2007) *Clin. Exp. Allergy,* 37: 794-802. | Patients & HCPs consider that mobile monitoring technology had potential to facilitate self-management.  Automated feedback could improve compliance with monitoring.  Scepticism as to whether electronic system could really change behaviour.  Some patients concerned about ‘ill model’.  Perceived advantages in electronically submitted models. |
|  |  | Van der Meer (2010) *Respir Res,* 11, 74. | Improvements in asthma control in intervention group (self-management guided by weekly self-monitoring) driven by improvements in patients with uncontrolled asthma. |
|  |  | Anhoj (2004). *J Med Internet Res,* 3, 6, e23. doi:[10.2196/jmir.6.3.e23](http://doi.org/10.2196/jmir.6.3.e23) | Patients usually stopped using symptom monitoring diary after a brief time (even after positive feedback).  Doctors reluctant to introduce diary to patients because of time constraints.  Patients were reluctant to increase medication when told to by ‘red alert’ as contradicted previous experience.  Patients ‘inherent attitudes’ were not changed by internet information.  Feedback messages (e.g. red alert) were often ignored.  Peak flow measurements detracted from diary useage.  Some users found website cumbersome and would have preferred mobile option. |
|  |  | Ure (2011) *Prim Car Resp J.,* | Telemonitoring in COPD viewed as non-intrusive access to professional care.  Patients considered it to recognise symptom exacerbation earlier.  Clinicians worried about increased workload, overtreating. |
|  |  | Rasmussen (2005), *J Allergy Clin Immunol,* 115, 6: 1137-1142. [doi:10.1016/j.jaci.2005.03.030](http://dx.doi.org/10.1016/j.jaci.2005.03.030) | Treatment with HCP managed online tool lead t orange of improvements including ACQ and AQLQ as well as lung function. |
| Patients may not be aware of / not acknowledge psychological aspects of condition, including contribution to symptoms and quality of life. | 1. Not aware of psychological aspects.  - Contribution to symptoms | Thomas (2015) *Prim Care Resp. Med.* 25: 15004. doi:[10.1038/npjpcrm.2015.4](http://www.nature.com/articles/npjpcrm20154) | Need for personalized medicine.  Role of anxiety and depression in disease. |
|  |  | **Thomas (2011) *Prim Care Respir J,* 20, 3: 250-256.** [**doi: 10.4104/pcrj.2011.00058.**](http://www.ncbi.nlm.nih.gov/pubmed/21674122) | **Primary care clinicians treating asthma should be aware of the possibility of psychological dysfunction in asthmatics, particularly those with poor control.** |
|  |  | Leander (2014). *Resp. Med,* 108, 11: 1594-1600. [doi:10.1016/j.rmed.2014.09.007](http://dx.doi.org/10.1016/j.rmed.2014.09.007) | Anxiety and depression related to wheezing, breathlessness and nightly symptoms. |
|  |  | Deshmukh (2007), *Resp Med,* 101, 2: 194-202. [doi:10.1016/j.rmed.2006.05.005](http://dx.doi.org/10.1016/j.rmed.2006.05.005). | Clinical anxiety and panic manifestations affect symptom perception and asthma management through the effects of anxiety symptoms such as hyperventilation, and indirectly through self-management behavior and physician response |
|  |  | De Peuter (2004). *Clin Psy Rev,* 24, 5, 557-581. [doi:10.1016/j.cpr.2004.05.001](http://dx.doi.org/10.1016/j.cpr.2004.05.001) | ‘Poor perceivers’ do notice changes in the state of their airways but do not interpret these sensations as such. |
|  |  | Jaen (2014). *J Psychosom Res,* 77, 4: 302-308. [doi:10.1016/j.jpsychores.2014.07.002](http://dx.doi.org/10.1016/j.jpsychores.2014.07.002) | Increased risk perception leads to increased symptom reporting. |
|  |  | Ritz (2014). *Allergy & Asthma Pro.* 35, 5: 390-397. [doi:10.2500/aap.2014.35.3779](http://www.ncbi.nlm.nih.gov/pubmed/25295806). | Perceived psychological asthma triggers explained up to 42.5% of the variance in asthma control and symptoms. |
|  |  | Janssens (2013) *Clin Exp Allergy, 43, 9*:1000-1008. [doi:10.1111/cea.12138](http://www.ncbi.nlm.nih.gov/pubmed/23957335) | Interventions that target accuracy of asthma trigger identification enhance asthma control.  Correlation between self-reported asthma triggers and allergy tests is only modest. |
|  |  | Yardley (2001). *J Anx Disorders,* 15, 1: 107-119. [doi:10.1016/S0887-6185(00)00045-1](http://dx.doi.org/10.1016/S0887-6185(00)00045-1). | Anxiety arousal and hyperventilation can increase somatic symptoms (in balance disorder).  Need to combine physiotherapy and psychotherapy for dizziness. |
|  |  | Kirby (2009). *J Psychosom Res.* 6, 2: 111-118. [doi:10.1016/j.jpsychores.2008.05.027](http://dx.doi.org/10.1016/j.jpsychores.2008.05.027) | Uncertainty of outcome (and less understanding of illness) increased anxiety (in Meniere’s disease) |
|  | 1. Not aware of impact on quality of life. | Koinis-Mitchell (2009). *Health Psyc,* 28, 2: 226-237.  [doi:10.1037/a0013169](http://psycnet.apa.org/journals/hea/28/2/226/) | Attentional abilities in children had more of a bearing on symptom monitoring accuracy than anything else. |
|  |  | Vazquez (2010). *J. Psychosom Res, 68, 2: 175-181. doi:* | More severe asthma symptoms related to higher trait anxiety, with no difference in self-management variables. |
|  |  | **Yorke (2007), *Resp Med, 101, 1:* 1-14.** [**doi:10.1016/j.rmed.2006.04.003**](http://dx.doi.org/10.1016/j.rmed.2006.04.003)**.** | **Recommendation that larger and well-conducted randomized trials use valid outcome measures to evaluate the effectiveness of psychological interventions for adults with asthma.** |
|  |  | Van Lieshout (2012). *Chem Immunol Allergy,* 98, 1-13. [*doi:10.1159/000336493*](http://www.ncbi.nlm.nih.gov/pubmed/22767054)*.* | Central cognitive processes influence interpretation of asthma symptoms. |
|  |  | Lehrer (2002) *J Consult Clin Psychol,* 70, 3 : 691-711. doi:[10.1037/0022-006X.70.3.691](http://psycnet.apa.org/doi/10.1037/0022-006X.70.3.691) | Promise in psychological interventions for asthma.  Defensiveness often associated with stress-related bronchoconstriction. |
|  |  | Al-Khateeb (2015) *Multidiscp Respir Med,* 10, 1: 15. doi:[10.1186/s40248-015-0011-6](http://dx.doi.org/10.1186%2Fs40248-015-0011-6) | QoL significantly adversely affected by asthma  Asthma significantly increased behavioural and emotional problems. |
|  |  | Hasler (2005) *Am J Respir Crit Care Med,* 171, 11: 1224-1230[. PMID:15764721](http://www.ncbi.nlm.nih.gov/pubmed/15764721) | Asthma predicted panic but panic did not predict asthma. |
